# Weight-adjusted waist index and cardiovascular disease: a population-based study in Ravansar, Iran

**DOI:** 10.1186/s13690-024-01451-w

**Published:** 2024-11-20

**Authors:** Sepehr Sadafi, Ali Azizi, Ebrahim Shakiba, Yahya Pasdar

**Affiliations:** 1 Clinical Research Development Center, Imam Reza Hospital, Kermanshah University of Medical Sciences, Kermanshah, Iran; 2 Social Development and Health Promotion Research Center, Kermanshah University of Medical Sciences, Kermanshah, Iran; 3 Department of Community and Family Medicine, School of Medicine, Kermanshah University of Medical Sciences, Kermanshah, Iran; 4 Research Center for Environmental Determinants of Health (RCEDH), Health Institute, Kermanshah University of Medical Sciences, Kermanshah, Iran

**Keywords:** Cardiovascular diseases, Obesity, Weight-adjusted-waist index, Persian

## Abstract

**Background:**

The weight-adjusted-waist index (WWI) is a relatively new index to obesity. This study aimed to explore the reationship between WWI and cardiovascular disease (CVD).

**Methods:**

This cross-sectional study included 8,899 participants aged 35 to 65 from the Ravansar non-communicable diseases (RaNCD) cohort study in Ravansar, Iran. The WWI was calculated by dividing waist circumference (WC) by the square root of weight. The receiver operating characteristic (ROC) curve was utilized to assess the predictive performance of WWI in relation to CVD. The study applied multiple logistic regression to assess the association between WWI and CVD.

**Results:**

Participants had an average age of 47.52 ± 8.29 years, with 45.30% being men and 41.13% residing in rural areas. The prevalence of CVD was found to be 17.36%. A positive correlation between WWI and CVD was obseved, with individuals in the highest WWI quartile having a 36% (OR = 1.36, 95%CI: 1.11, 1.78) greater odds of CVD compared to those in the lowest quartile (OR = 1.03, 95%CI: 0.79, 1.33) (ptrend = 0.010). Subgroup analyses indicated stronger links between WWI and CVD among participants over 50, males, urban residents, those of high socioeconomic status (SES), and passive smokers (*p* < 0.001). The ROC analysis revealed that WWI is a greater ability in predicting CVD (AUC: 0.64, 95%CI: 0.61, 0.64) compared to body mass index (BMI) (AUC: 0.60, 95%CI: 0.58, 0.61) and WC (AUC: 0.61, 95%CI: 0.59, 0.62).

**Conclusion:**

The increase in WWI elevates the odds of CVD, making the management of WWI crucial for CVD prevention.

**Table:** 

Text box 1. Contributions to the literature	
- This research presents an innovative tool for evaluating cardiovascular risk, improving upon current assessment methods. - It offers population-based data that connects adjustments in waist circumference to better predictions of cardiovascular disease. - These results add to the expanding literature that supports the use of creative strategies in public health evaluations of cardiovascular wellness.	

## Introduction

Obesity represents a significant risk factor for numerous chronic metabolic conditions by health system [1–3]. Projections indicate that by 2030, approximately 38% of adults globally will be classified as obese [4]. A meta-analysis by Wong et al. (2020) reported a global prevalence of central obesity at 41.5% [5]. Previous studies have indicated that obesity increases the risk of cardiovascular disease (CVD) [6, 7]. However, conflicting findings from some studies suggest that overweight and obese individuals may have a lower risk of CVD and hypertension compared to those with normal weight, and being overweight or obese may play a protective role [8–10]. A study conducted in 2020 found that the average risk of developing cardiovascular disease over a 10-year period was 16.4% [11].

Among various anthropometric indices, body mass index (BMI) and waist circumference (WC) are commonly utilized as key indicators for assessing both general and central obesity [12]. Unlike BMI, which reflects general obesity, WC specifically measures total body fat [13]. Park et al. (2018) introduced the weight-adjusted-waist index (WWI) as a novel measure of obesity [14]. This index evaluates central obesity by considering both muscle and fat mass, calculated by dividing WC by the square root of body weight [15, 16]. Several studies have reported a positive association between WWI and chronic diseases such as CVD, stroke, non-alcoholic fatty liver disease (NAFLD) and chronic kidney disease [17–20]. However, a comprehensive study in this area has yet to be conducted in Iran.

CVD is a chronic condition influenced by multiple factors [21], with obesity recognized as a significant risk factor. Therefore, reducing obesity can be effective in the prevention and management of CVD. WWI may serve as a more sensitive measure for identifying individuals at risk for CVD associated with obesity, particularly in groups with diverse body fat levels. Hence, the primary objective of this study was to examine the relationship between WWI and CVD. The secondary objective was to evaluate how the predictive capabilities of WWI compare to those of BMI and WC among adults in western Iran.

## Methods

### Data sources and participants

**Fig. 1:**
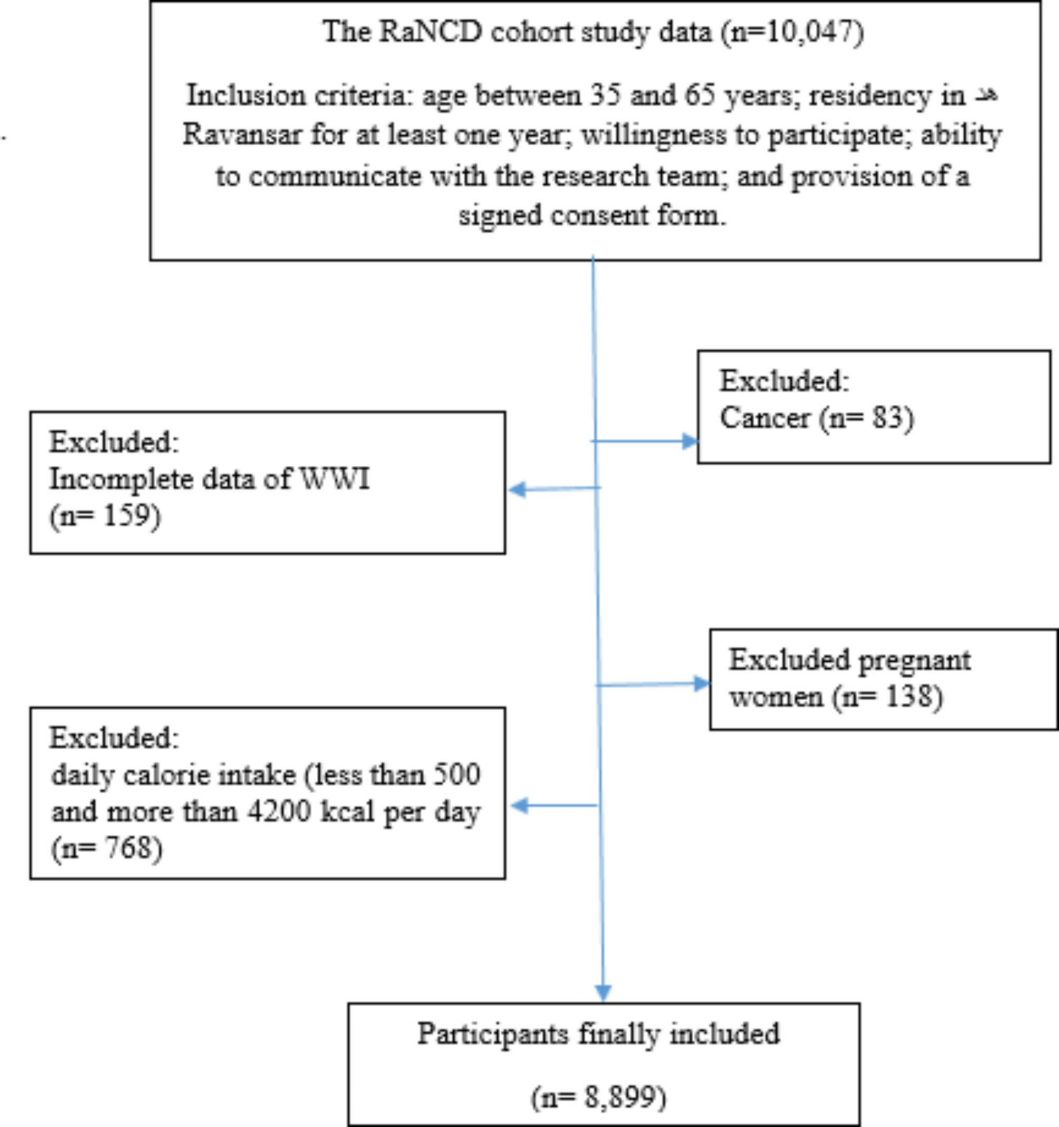
Flowchart of the study participants Abbreviation: RaNCD: Ravansar non-communicable diseases

This study is a cross-sectional analysis of data from the baseline phase of the Ravansar non-communicable diseases (RaNCD) cohort study [22]. The RaNCD cohort is one of the Prospective Epidemiological Research Studies in Iran (PERSIAN) [23]. The baseline phase of the RaNCD study began in 2014 with a 15-year design and involved 10,047 adults aged 35 to 65 living in both urban and rural areas of Ravansar, western Iran. The participants were predominantly of Kurdish ethnicity. The study profile for the RaNCD cohort has been published previously, providing additional details about the study’s design and methodology [22]. All participants from the initial phase of the RaNCD cohort were involved in this research (*n* = 10,047). Following the application of exclusion criteria, 8,899 participants were examined (Fig. 1).

### Study variables

The data was collected in compliance with the cohort study protocol, and trained professionals used digital questionnaires to gather information. The socio-economic status (SES) was established by considering factors such as education level, place of residence, welfare amenities and wealth through the principal component analysis (PCA) method. After this analysis, the SES was divided into three categories (low, moderate, and high) [24]. Physical activity was assessed using 22 questions related to sports, work, and leisure activities over a 24-hour period, measured in Metabolic Equivalent of Task per hour per day (MET/hour per day). It was classified into three categories: low (24–36.5 MET/hour per day), moderate (36.6–44.4 MET/hour per day), and high (≥ 44.5 MET/hour per day) [25]. The standard of National Health Interview Survey (NHIS) current smoking definition, which screens for lifetime smoking ≥ 100 cigarettes, was used. Exposure to cigarette smoke at home, in the workplace, etc., is defined as passive smoking in people who are not smokers themselves. Participants were classified as former smokers if they had not smoked regularly or occasionally in the past year and had smoked more than 100 cigarettes in their lifetime were classified as former smokers [26, 27].

The anthropometric measurements such as BMI, percent body fat (PBF), fat mass index (FMI), waist-hip ratio (WHR), WC, visceral fat area (VFA), skeletal muscle mass (SMM) and total body weight (TBW) were determined using an Impedance Analyzer BIA (Inbody 770, Inbody Co, Seoul, Korea). In the RaNCD cohort study, BMI, WC, WHR and Hip Circumference (HC) indicators were measured twice—once using a BIA and once with a centimeter and scale, by standard measurement methods due to their sensitivity. A BMI between 25 and 30 is classified as overweight, while a BMI greater than 30 kg/m² is categorized as general obesity [22]. The dietary intake of participants was assessed using the food frequency questionnaire (FFQ) [28].

The lipid profile, consisting of triglycerides (TG), total cholesterol (TC), high-density lipoprotein cholesterol (HDL-C), low-density lipoprotein cholesterol (LDL-C), and fasting blood sugar (FBS), was assessed by drawing 25 cc of blood from the participants. As per the protocol, participants were instructed to fast for 8–12 h before the blood collection.

The WWI was computed by dividing the waist circumference (cm) by the square root of the body weight (kg) [14]. A patient with CVD is defined as someone who has experienced at least one of the following conditions: a history of ischemic heart disease (IHD), heart failure, angina, stroke, myocardial infarction (MI), or is currently using medication for CVD. This definition is based on the International Statistical Classification of Diseases and Related Health Problems (ICD-10). The specific type of CVD is determined through a cardiologist’s diagnosis and classified using the appropriate ICD-10 code. The participants’ systolic and diastolic blood pressure (SBP and DBP) were assessed using the standard method while seated on a chair, following a 10-minute rest period, with measurements taken from both the right and left arms [29]. Subsequently, the average was computed. Participants with SBP ≥ 140mmHg and/or DBP ≥ 90mmHg, or those taking antihypertensive medications, were classified as hypertensive [29].

### Statistical analysis

In this study, Stata software version 14.2 (Stata Corp, College Station, TX, USA) was used to perform all analyses. The study was presented the basic characteristics of the participants as mean ± standard deviation (SD) and number (percentage) across WWI quartiles. To calculate the average of the food groups, we adjusted the calorie intake and presented the findings as the mean ± standard error (SE). The normality of the data was assessed using the Kolmogorov-Smirnov test. We used the one-way ANOVA test for continuous variables and the chi-square test for qualitative variables to compare the differences among WWI quartiles. Logistic regression analysis was conducted to explore the association between CVD and WWI. The multiple regression model was adjusted for age, sex, residence, SES, smoking, physical activity, hypertension, energy intake, FBS, LDL, HDL, TG and TC variables. Receiver operating characteristic (ROC) curve analysis was conducted to evaluate the predictive ability of WWI, WC, and BMI for CVD. This analysis used the area under the curve (AUC) along with a 95% confidence interval to assess the effectiveness of these measurements. Throughout the analyses, a p-value of less than 0.05 with 95% confidence intervals (CIs) was deemed significant.

## Results

### Participant characteristics

The average age of the participants was 47.52 ± 8.29 years, with 45.30% being men and 41.13% residing in rural areas. Among the 8,899 participants, 1,545 (17.36%) had CVD. Participants in the highest quartile of the WWI were significantly older than those in the lowest quartile (43.90 ± 7.16 vs. 51.10 ± 8.38, ptrend < 0.001). The prevalence of hypertension and CVD increased significantly across WWI quartiles (ptrend < 0.001). Additionally, vigorous physical activity levels were lower in the fourth WWI quartile compared to the first quartile (ptrend < 0.001). The average FBS, LDL-C, HDL-C and TC also rose significantly across WWI quartiles (ptrend < 0.001) (Table 1).

**Table 1:** Baseline characteristics of study participants categorized by weight-adjusted waist index quartiles during the baseline phase (2015) of the Ravansar non-communicable diseases (RaNCD) cohort study conducted in Ravansar, Iran (*n* = 8899)

Variables	Total (*n* = 8899)	Weight-adjusted-waist index				*P* value trend*
		Q1 (*n* = 2114)	Q2 (*n* = 2195)	Q3 (*n* = 2281)	Q4 (*n* = 2309)	
WWI (Min, Max)	7.17, 16.06	7.17,10.88	10.89,11.46	11.47, 12.05	12.06, 16.06	
Age, (year), mean ± SD	47.52 ± 8.29	43.90 ± 7.16	46.39 ± 7.76	48.34 ± 8.04	51.10 ± 8.38	< 0.001
Sex, n (%)						
Men	4031 (45.30)	1578 (39.15)	1283 (31.83)	884 (21.93)	286 (7.10)	< 0.001
Women	4868 (54.70)	536 (11.01)	912 (18.73)	1397 (28.70)	2023 (41.56)	
Residence, n (%)						
Urban	5239 (58.87)	1535 (29.30)	1378 (26.30)	1222 (23.33)	1104 (21.07)	0.001
Rural	3660 (41.13)	579 (15.82)	817 (22.32)	1059 (28.93)	1205 (32.92)	
Socioeconomic status (MET/h/day), n (%)						
Low	2994 (33.66)	458 (21.67)	617 (28.12)	840 (36.83)	1079 (46.77)	< 0.001
Moderate	2954 (33.21)	683 (32.31)	714 (32.54)	779 (34.15)	778 (33.72)	
High	2948 (33.14)	973 (46.03)	863 (39.33)	662 (29.02)	450 (19.51)	
Smoking, n (%)						
Never	3712 (41.92)	764 (36.33)	906 (41.56)	963 (42.37)	1079 (46.91)	< 0.001
Current	966 (10.91)	360 (17.12)	290 (13.30)	221 (9.72)	95 (4.13)	
Former	740 (8.36)	191 (9.08)	195 (8.94)	192 (8.45)	162 (7.04)	
Passive	3438 (38.82)	788 (37.47)	789 (36.19)	897 (39.46)	964 (41.91)	
Physical activity (MET/h per day), n (%)						
Low	2736 (30.75)	677 (32.02)	717 (32.67)	639 (28.01)	703 (30.45)	< 0.001
Moderate	4298 (48.30)	850 (40.21)	970 (44.19)	1184 (51.91)	1294 (56.04)	
Vigorous	1865 (20.96)	587 (27.77)	508 (23.14)	458 (20.08)	312 (13.51)	
Hypertension, n (%)	1416 (15.91)	243 (11.49)	321 (14.62)	364 (15.96)	488 (21.13)	< 0.001
CVD, n (%)	1545 (17.36)	207 (9.79)	307 (13.99)	413 (18.11)	618 (26.76)	< 0.001
FBS (mg/dL), mean ± SD	97.10 ± 29.64	92.39 ± 24.34	96.24 ± 29.30	98.73 ± 32.16	100.60 ± 31.18	< 0.001
TG (mg/dL), mean ± SD	136.76 ± 81.58	139.42 ± 87.48	135.65 ± 81.74	134.66 ± 78.23	137.47 ± 78.98	0.551
LDL-C (mg/dL), mean ± SD	111.66 ± 31.34	108.11 ± 30.03	109.31 ± 29.08	112.33 ± 30.90	116.49 ± 33.64	< 0.001
HLD-C (mg/dL), mean ± SD	46.60 ± 11.36	43.44 ± 10.58	45.14 ± 10.76	47.66 ± 11.35	49.83 ± 11.62	0.001
TC (mg/dL), mean ± SD	185.61 ± 37.74	179.44 ± 36.12	181.57 ± 35.93	186.91 ± 36.79	193.82 ± 40.51	< 0.001
SBP (mmHg), mean ± SD	108.11 ± 17.03	107.51 ± 16.01	108.08 ± 16.66	107.68 ± 17.05	109.10 ± 18.19	0.092
DBP (mmHg), mean ± SD	69.73 ± 9.90	70.30 ± 9.72	70.08 ± 10.08	69.17 ± 9.76	69.44 ± 10.01	< 0.001

* *P*-value was obtained one-way ANOVA and Chi square tests

Abbreviation: HDL-C: High-density lipoprotein cholesterol, LDL-C: Low-density lipoprotein cholesterol, TG: Triglycerides, TC: Total cholesterol, FBS: Fasting blood sugar, CVD: cardiovascular diseases; Q: quartile

### Anthropometric indices and dietary intake of the study participants

Table 2 outlines the anthropometric indicators and dietary intake of participants based on WWI quartiles. Those in the highest WWI quartile exhibited higher values for BMI, WC, WHR, FMI, VFA and PBF compared to the lowest quartile (ptrend < 0.001). The average SMM in the first quartile was significantly higher than that in the fourth quartile (29.61 ± 5.70 vs. 22.32 ± 3.62, ptrend < 0.001). Furthermore, TBW was lower in the highest WWI quartile (Q1: 39.34 ± 6.92 vs. Q4: 30.25 ± 4.37, ptrend < 0.001). The fourth quartile of WWI had the highest percentage of energy intake from carbohydrates, while the first quartile had the highest energy intake from protein.

**Table 2:** Anthropometric indices and dietary intake of the study participants according to weight-adjusted-waist index quartiles in the participants of the baseline phase (2015) of the Ravansar non-communicable diseases (RaNCD) cohort study conducted in Ravansar, Iran (*n* = 8899)

Variables	Weight-adjusted-waist index				*P* value trend*
	Q1 (*n* = 2114)	Q2 (*n* = 2195)	Q3 (*n* = 2281)	Q4 (*n* = 2309)	
**Anthropometric indices**					
WWI, mean ± SD	10.40 ± 0.42	11.18 ± 0.17	11.75 ± 0.17	12.52 ± 0.37	< 0.001
BMI (kg/m^2^), mean ± SD	26.58 ± 4.36	27.07 ± 4.50	27.31 ± 4.48	28.76 ± 4.90	< 0.001
WHR, mean ± SD	0.93 ± 0.06	0.94 ± 0.06	0.94 ± 0.06	0.95 ± 0.06	< 0.001
WC (cm), mean ± SD	90.13 ± 9.20	95.58 ± 8.83	98.56 ± 8.80	104.10 ± 10.10	< 0.001
SMM (kg), mean ± SD	29.61 ± 5.70	27.21 ± 5.36	25.10 ± 4.54	22.32 ± 3.62	< 0.001
FMI (kg/m^2^), mean ± SD	7.94 ± 3.51	8.97 ± 3.77	9.85 ± 3.77	11.87 ± 3.87	< 0.001
VFA (cm^2^), mean ± SD	102.73 ± 45.80	114.64 ± 50.22	123.81 ± 49.97±	147.77 ± 48.94	< 0.001
PBF (%), mean ± SD	28.80 ± 8.58	32.01 ± 9.11	34.95 ± 8.74	40.25 ± 7.48	< 0.001
TBW (liter), mean ± SD	39.34 ± 6.92	36.53 ± 6.50	33.58 ± 5.55	30.25 ± 4.37	< 0.001
**Dietary intake**					
Energy (kcal/d), mean ± SD	2792.39 ± 718.69	2622.03 ± 720.97	2476.39 ± 719.08	2224.99 ± 690.78	< 0.001
Carbohydrate (%E)	61.10 ± 6.10	61.46 ± 6.10	61.56 ± 6.21	61.70 ± 6.35	0.002
Fat (%E)	26.88 ± 5.94	26.62 ± 5.98	26.79 ± 5.89	26.85 ± 6.09	0.613
Protein (%E)	14.03 ± 2.26	13.83 ± 2.11	13.60 ± 2.10	13.48 ± 2.09	< 0.001
Bread and cereals (g/d), mean ± SE	520.49 ± 3.47	523.99 ± 3.38	508.03 ± 3.27	506.45 ± 3.31	0.001
Fruits (g/d), mean ± SE	264.70 ± 4.43	259.83 ± 4.31	269.18 ± 4.18	266.51 ± 4.23	0.397
Vegetables (g/d), mean ± SE	270.31 ± 3.51	268.68 ± 3.55	266.84 ± 3.45	269.07 ± 3.48	0.910
Dairy (g/d), mean ± SE	371.58 ± 8.01	427.60 ± 7.78	472.84 ± 7.54	482.39 ± 7.63	0.001
Legumes (g/d), mean ± SE	36.10 ± 0.66	32.17 ± 0.64	31.98 ± 0.62	31.24 ± 0.63	0.001
Red meat (g/d), mean ± SE	22.22 ± 0.56	20.50 ± 0.57	20.30 ± 0.56	18.81 ± 0.56	0.004
White meat (g/d), mean ± SE	53.31 ± 0.86	51.03 ± 0.84	48.24 ± 0.80	48.11 ± 0.82	< 0.001
Egg, mean ± SE	23.59 ± 0.40	21.14 ± 0.39	19.02 ± 0.38	17.01 ± 0.38	< 0.001
Sweets & desserts, mean ± SE	57.10 ± 0.79	55.91 ± 0.76	58.90 ± 0.74	57.31 ± 0.76	0.215
Tea & coffee, mean ± SE	700.27 ± 10.57	702.32 ± 10.23	733.58 ± 10.01	727.04 ± 10.17	0.046
Nuts, mean ± SE	9.05 ± 0.21	7.98 ± 0.21	7.92 ± 0.20	8.30 ± 0.21	< 0.001

*P-value was obtained one-way ANOVA test

The mean ± SE of dietary intake is adjusted for daily energy intake

Abbreviation: BMI: Body mass index; TBW: Total body water; VFA: Visceral fat area; PBF: Percent body fat; WC: Waist circumference; WHR: Waist hip ratio; SMM: Skeletal muscle mass; FMI: Fat mass index; Q: quartile

### Association between WWI and CVD

The unadjusted model indicates that the odds of CVD significantly increase with higher WWI in both men and women (ptrend < 0.001). After adjusting for age, sex, and residence, the odds of developing CVD increased by 1.07 times (OR: 1.07, 95% CI: 0.88, 1.31) in the second quartile, 1.13 times (OR: 1.13, 95% CI: 0.93, 1.38) in the third quartile, and 1.26 times (OR: 1.26, 95% CI: 1.04, 1.54) in the fourth quartile of WWI (ptrend = 0.025).

Further adjustments for additional factors such as SES, smoking, physical activity, hypertension, energy intake, FBS, LDL, HDL, TG, TC revealed that the odds of developing CVD increased by 1.03, 1.25, and 1.36 times in the second, third, and fourth WWI quartiles, respectively, compared to the first quartile (ptrend = 0.010) (Table 3).

**Table 3:** The association between weight-adjusted-waist index and cardiovascular diseases among participants of the baseline phase (2015) of the Ravansar non-communicable diseases (RaNCD) cohort study conducted in Ravansar, Iran

Weight-adjusted-waist index quartiles	Model 1	Model 2	Model 3
	OR (95% CI)		
**Q1**	Ref (1.00)	Ref (1.00)	Ref (1.00)
**Q2**	1.50 (1.24, 1.81)	1.07 (0.88, 1.31)	1.03 (0.79, 1.33)
**Q3**	2.03 (1.70, 2.43)	1.13 (0.93, 1.38)	1.25 (0.98, 1.61)
**Q4**	3.37 (2.83, 3.99)	1.26 (1.04, 1.54)	1.36 (1.11, 1.78)
**P trend**	< 0.001	0.025	0.010

Abbreviation: OR: odds ratio; CI: confidence interval

Model 1: Unadjusted; Model 2: Adjusted for age, sex and residence; Model 3: Adjusted for age, sex, residence, socio economic status, smoking, physical activity, hypertension, energy intake, high-density lipoprotein cholesterol, low-density lipoprotein cholesterol, triglycerides, total cholesterol, fasting blood sugar

### Association between WWI and CVD by subgroup analysis

Table 4 presents subgroup analyses stratified by age, sex, place of residence, SES, smoking, hypertension, physical activity, and obesity to assess the association between WWI and CVD.

**Table 4:** Subgroup analysis of the association between weight-adjusted-waist index and cardiovascular diseases among participants of the baseline phase (2015) of the Ravansar non-communicable diseases (RaNCD) cohort study conducted in Ravansar, Iran

Subgroup	OR (95% CI) *	*P* value
Age		
35–50 years	1.22 (1.04, 1.45)	0.022
51–65 years	1.26 (1.10, 1.46)	0.002
Sex		
Men	1.24 (1.02, 1.59)	0.036
Women	1.14 (1.02, 1.10)	0.048
Residence		
Urban	1.17 (1.03, 1.33)	0.025
Rural	1.21 (0.98, 1.49)	0.069
Socioeconomic status		
Low	1.10 (0.92, 1.31)	0.312
Moderate	1.19 (0.98, 1.45)	0.072
High	1.30 (1.04, 1.63)	0.020
Smoking		
Never	1.13 (0.95, 1.35)	0.164
Current	0.90 (0.60, 1.34)	0.614
Former	1.26 (0.89, 1.78)	0.184
Passive	1.28 (1.10, 1.53)	0.007
Physical activity (MET/h per day)		
Low	1.15 (0.93, 1.40)	0.180
Moderate	1.23 (1.06, 1.44)	0.008
Vigorous	1.10 (0.82, 1.45)	0.530
Hypertension		
No	1.18 (1.03, 1.36)	0.017
Yes	1.14 (0.94, 1.36)	0.172
Body mass index		
< 25 kg/m^2^ (normal)	1.18 (1.02, 1.40)	0.023
≥ 25 kg/m^2^ (overweight &obese)	1.10 (0.92, 1.32)	0.286

Abbreviation: OR: odds ratio; CI: confidence interval

*Adjusted for age, sex, residence, socio economic status, smoking, physical activity, hypertension, energy intake, high-density lipoprotein cholesterol, low-density lipoprotein cholesterol, triglycerides, total cholesterol, fasting blood sugar

A positive correlation was found between WWI and CVD in two age groups, with a stronger association in the 51 to 65-year-old group (OR: 1.26, 95% CI: 1.10, 1.46). Additionally, for each unit increase in WWI, the odds of CVD rose by 24% in men and 14% in women (*p* < 0.05). While the odds of CVD increased with WWI in both urban and rural areas, the increase was not statistically significant in rural settings.

### Comparing the predictive power of WWI with BMI and WC for CVD using ROC curve analysis

**Fig. 2:**
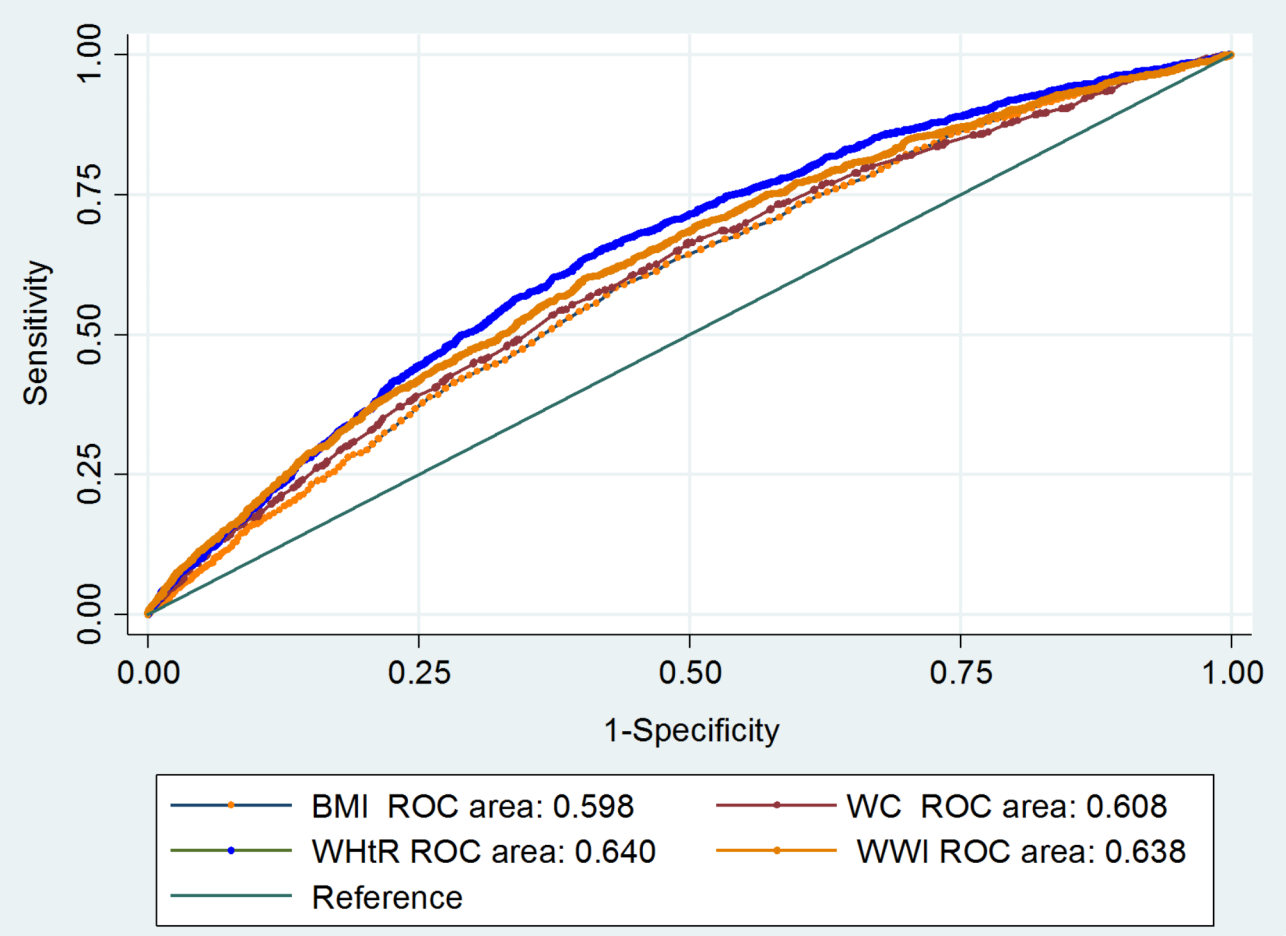
ROC curve analysis of weight-adjusted-waist index (WWI), body mass index (BMI), waist-to-height ratio (WHtR) and waist circumference (WC) for predicting of cardiovascular diseases among participants of the baseline phase (2015) of the Ravansar non-communicable diseases (RaNCD) cohort study conducted in Ravansar, Iran

The results indicated that WWI had a superior predictive capability for CVD (AUC: 0.64, 95% CI: 0.61, 0.64) compared to BMI (AUC: 0.60, 95% CI: 0.58, 0.61) and WC (AUC: 0.61, 95% CI: 0.59, 0.62) in the studied population (Fig. 2).

## Discussion

In this cross-sectional study involving 8,899 Iranian adults, an increase in WWI was found to elevate the odds of CVD. Specifically, the odds of developing CVD in the second, third, and fourth quartiles of WWI were 1.03, 1.25, and 1.36 times higher than in the first quartile, respectively. Furthermore, subgroup analyses revealed a higher correlation between WWI and CVD among participants over 50 years old, males, urban residents, those with high SES, and passive smokers. The ROC analysis demonstrated that WWI had superior predictive capability for CVD compared to BMI and WC in the study population.

A cross-sectional study of U.S. adults found a positive association between WWI and CVD [17]. Another study involving 23,389 participants from the National Health and Nutrition Examination Survey (NHANES) also showed a significant increase in stroke prevalence with rising WWI [18]. These findings align with previous research, reinforcing the positive association between WWI and the odds of CVD.

The WWI primarily reflects central obesity independently of overall weight. It has shown greater accuracy compared to BMI [15, 30, 31]. Traditionally, changes in body weight were frequently utilized in the BMI formula to indicate fat accumulation and obesity in the adult population due to height stability. However, recent research has questioned this approach [32, 33]. Furthermore, with the introduction of the muscle-fat axis concept, researchers have noted that weight loss can also be associated with a reduction in muscle mass, often accompanied by an increase in visceral fat. This may suggest a more precise assessment of obesity [34]. Several non-traditional obesity indices, such as the Visceral Adiposity Index (VAI), Body Roundness Index (BRI), A Body Shape Index (ABSI), and Atherogenic Index of Plasma (AIP), have been found to effectively predict metabolic diseases, including CVD [35, 36]. Research by Hamzeh et al. involving 7,362 individuals demonstrated that VAI and AIP are reliable markers for predicting CVD [36]. However, calculating these indices can be complex and require invasive methods.

Recent studies indicate that WWI is a better predictor of metabolic disease risk than BMI and WC [19, 37, 38]. Additionally, body composition indicators have proven to be as effective as, or a suitable alternative to, traditional anthropometric measures for predicting non-communicable diseases (NCDs) [35, 39]. In the current study, ROC curve analysis revealed that WWI has greater predictive power for CVD compared to BMI and WC. Overall, WWI is straightforward, cost-effective, and practical to compute, highlighting its efficacy in forecasting CVD odds, which should be considered by healthcare providers in health systems. The subgroup analyses identified a more robust link between WWI and CVD among individuals aged over 50 years old. In contrast, two previous studies indicated that this association was stronger in younger individuals (under 50), attributing it to higher obesity rates in that age group [17, 18]. This discrepancy could be explained by the nearly equal prevalence of obesity in both age groups in the current study.

The multivariate regression analysis did not reveal any relationship between physical activity and CVD. However, there was a notable decreasing trend in the level of intense physical activity across WWI quartiles, which is an important finding. Generally, as societies industrialize and urbanize, physical activity tends to decline, leading to increased general and central obesity, and consequently, a rise in cardiometabolic diseases [40, 41]. Interestingly, this study found that the association between heart disease and weight was stronger among urban residents compared to those living in rural areas.

A meta-analysis conducted in 2020 found that passive smoking is significantly associated to a higher risk of CVD incidence and mortality [42]. In our study, the association between WWI and CVD was 26% greater in passive smokers compared to non-smokers. The reason for this increased odd in passive smokers versus current smokers remains uncertain. On the other hand, due to the inconsistent results regarding the connection between obesity and smoking [26, 43, 44], it is crucial to conduct longitudinal studies in diverse population subgroups to better understand this relationship.

In this study, we found that individuals in the higher WWI quartiles derived more of their energy intake from carbohydrates and less from protein. Additionally, those in the fourth quartile consumed more red meat compared to the first quartile, while their intake of white meat and nuts was lower. A systematic review and dose-response meta-analysis indicated that higher total protein intake is linked to a reduced risk of all-cause mortality, with a 5% decrease in mortality risk for each 3% increase in energy from vegetable proteins daily [45]. However, there is a positive correlation between dietary intake and obesity [46]. Furthermore, increased daily calorie intake, particularly from fats, has been associated with hypertension, dyslipidemia, and CVD [47, 48]. To explore the relationship between heart disease and weight across different dietary patterns and calorie intakes, longitudinal studies conducted by nutritionists are necessary.

The relationship between the WWI and CVD can be attributed to several mechanisms. Central obesity is known to increase oxidative stress in the body, which is closely associated with the development of atherosclerosis [49, 50]. A higher WWI indicates an accumulation of excess body fat and a decrease in muscle mass. This imbalance disrupts the release of adipocytokines, triggers inflammatory responses, causes endothelial dysfunction, and reduces physical function, all of which contribute to the onset of CVD [51–53]. Furthermore, metabolic conditions linked to obesity like glucose intolerance, hypertriglyceridemia, and hypertension also heighten the risk of CVD, particularly stroke [54].

This study has several strengths. Firstly, it utilized a national sample of the general adult population in Western Iran from the RaNCD study, which adhered to strict protocols and quality controls [22]. Secondly, we adjusted for most potential confounding variables to enhance the accuracy of the results. However, we cannot claim to have eliminated the influence of all possible confounders. There are also limitations to this study; the cross-sectional design prevents us from establishing a direct cause-and-effect relationship between WWI and CVD. Therefore, further longitudinal studies are needed to confirm these results.

## Conclusion

The results of the current study indicate that the odds of developing CVD in Iranian adults significantly increase with WWI gain. A more significant correlation was noted among participants over 50 years old, males, urban residents, those with high SES, and passive smokers. Additionally, WWI was found to be the most effective obesity index for predicting CVD compared to BMI and WC in this population. Therefore, managing WWI is recommended to help prevent CVD in Iranian adults. However, further validation of these results is necessary through longitudinal and prospective studies. We suggest some intervention strategies aimed at preventing CVD, which include: providing nutrition education, promoting physical activity, and performing regular health screenings for obesity and cardiovascular risk factors, especially among high-risk groups such as individuals over 50, men, and urban residents. Additionally, it is crucial to educate healthcare providers about the importance of weight management for their patients and to equip them with effective tools to achieve this.

## Data Availability

The data analyzed in the study are available from the corresponding author upon reasonable request.
